# Optoelectronic performance of indium tin oxide thin films structured by sub-picosecond direct laser interference patterning

**DOI:** 10.1038/s41598-023-37042-y

**Published:** 2023-06-16

**Authors:** Herman Heffner, Marcos Soldera, Andrés Fabián Lasagni

**Affiliations:** 1 Institut Für Fertigungstechnik, Technische Universität Dresden, George-Bähr-Str. 3c, 01069 Dresden, Germany; 2 Departamento de Química, Universidad Nacional del Sur, Instituto de Química del Sur (INQUISUR, UNS-CONICET), Av. Alem 1253, B8000CPB Bahía Blanca, Argentina; 3 Fraunhofer-Institut Für Werkstoff Und Strahltechnik (IWS), Winterbergstr 28, 01277 Dresden, Germany

**Keywords:** Materials for optics, Surface patterning, Electronic devices, Optical materials and structures, Nonlinear optics, Sub-wavelength optics

## Abstract

A route to increase the efficiency of thin film solar cells is improving the light-trapping capacity by texturing the top Transparent Conductive Oxide (TCO) so that the sunlight reaching the solar absorber scatters into multiple directions. In this study, Indium Tin Oxide (ITO) thin films are treated by infrared sub-picosecond Direct Laser Interference Patterning (DLIP) to modify the surface topography. Surface analysis by scanning electron microscopy and confocal microscopy reveals the presence of periodic microchannels with a spatial period of 5 µm and an average height between 15 and 450 nm decorated with Laser-Induced Periodic Surface Structures (LIPSS) in the direction parallel to the microchannels. A relative increase in the average total and diffuse optical transmittances up to 10.7% and 1900%, respectively, was obtained in the 400–1000 nm spectral range as an outcome of the interaction of white light with the generated micro- and nanostructures. The estimation of Haacke’s figure of merit suggests that the surface modification of ITO with fluence levels near the ablation threshold might enhance the performance of solar cells that employ ITO as a front electrode.

## Introduction

Transparent Conductive Oxides (TCOs) are extensively employed in optoelectronic devices due to their ability to work not only as a top electrode but also as an optical window. Some of the applications are related to touch screens^1^, smart windows^2^, photodiodes^3^, OLEDs^4^, and solar cells, such as those based on perovskite^5–7^, thin-film inorganic^8,9^, Dye-Sensitized (DSSCs)^10^, and organic materials^11^. Among the different TCOs that are being employed in the optoelectronic industry, Indium Tin Oxide (ITO) stands out for its high transparency and low sheet resistance $({R}_{\mathrm{S}})$^12^ which are key attributes for producing highly-efficient solar cells^13–16^. To approach the maximum theoretical efficiency on solar cells dictated by the limit imposed by Shockley’s and Queisser’s theory^17^, different strategies have been studied to further improve the ITO surface, optical and electrical properties and thus reduce the associated absorption, reflectance and electrical losses. Some of them include increasing the total transmittance $({T}_{\mathrm{tot}})$ by a higher surface roughness^18^, enhancing the optical path and diffuse transmittance $({T}_{\mathrm{diff}})$ by creating regular surface patterns^19^, reducing the sheet resistance by increasing the grain size^20^ or changing the chemical composition using laser annealling^21^.

Regarding surface modification using laser techniques, Jiang et al.^22^ textured ITO thin films by employing an infrared (1030 nm) femtosecond laser source. The generated periodic transparent nanowires induced up to 197% relative increase in the average transmittance compared to untreated ITO, especially on the infrared 1200–2000 nm spectral region. However, as a result of the removal of conductive material, the resistivity of the laser-treated films increased linearly with the applied laser fluence. Similarly, Liu et al.^23^ fabricated Laser-Induced Periodic Surface Structures (LIPSS) on ITO thin films using a picosecond (10 ps) infrared (1064 nm) solid-state laser. The average total transmittance underwent a remarkable enhancement of ~ 100% (relative) in the 1200–1900 nm range. Nonetheless, the laser-treated ITO thin films experienced a three times higher electrical surface resistance compared to the untreated reference_._ This implies that a trade-off between transmittance improvement and the worsening of the electrical properties should be carefully considered when employing laser techniques to structure transparent electrodes.

Another method for producing textured surfaces is Direct Laser Interference Patterning (DLIP)^24^. In DLIP, two or more laser beams are overlapped on the sample surface to generate an interference pattern so that the material can be locally melted or ablated at the maxima positions of the periodic intensity distribution^25–27^. Several TCO materials have already been treated with this technique. For instance, Eckhardt et al.^28^ performed a two- and three-beam DLIP process on 900 nm thick Al-doped ZnO by employing a 355 nm nanosecond laser source to obtain line-like as well as hexagonal-like structures with ~ 2 µm of spatial period. An enhancement in the spread of light was obtained as a result of the generated periodic microstructures, leading to an increase of the diffuse transmittance up to 50% in the 300–800 nm spectral range. Nevertheless, a small increase of up to 11.3% in the sheet resistance was observed after the laser treatment. In other work, Ring et al.^29^ obtained U-shaped groove structures with a spatial period of 860 nm by employing two-beam DLIP on 450 nm thick Al-doped ZnO using a UV (355 nm) picosecond (10 ps) laser source. The resulting TCO was utilized as a front contact in a silicon-based tandem solar cell and achieved a 10% relative increase in efficiency compared to the cell built with unstructured electrodes. The mentioned efficiency increase was ascribed to an improved light-trapping produced by the microtextures.

Additional DLIP research about surface modification of TCOs was reported by other authors^30–33^. Nevertheless, these works were carried out employing UV laser sources, which tend to have long-term stability issues (especially at 266 nm) and higher costs in terms of laser power (€/W)^34–36^. To the best of our knowledge, only a few studies were published in which cost-effective and stable IR laser sources were employed to modify the optoelectronic characteristics of TCOs using the DLIP technique^37–40^. However, to date, no reports have shown ITO films structured by DLIP at that wavelength.

In the present investigation, ITO thin films are treated with a two-beam DLIP technique based on infrared (1030 nm) sub-picosecond laser radiation. The changes in surface topography induced by the laser radiation are analyzed, as well as the modification of the optical and electrical properties.

## Methodology

**Figure 1:**
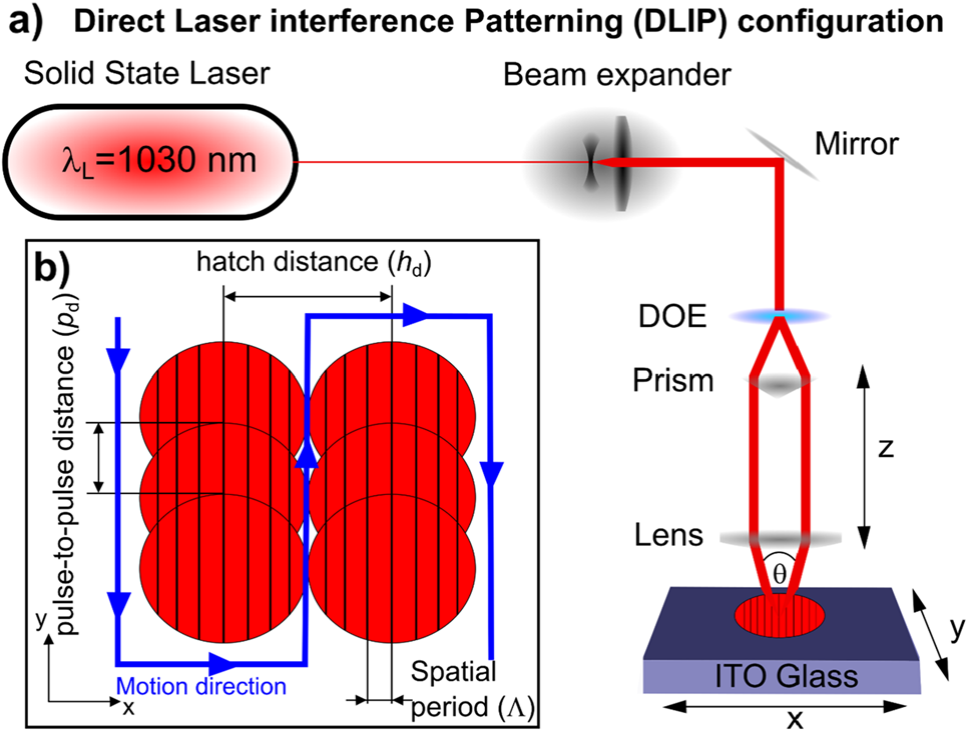
(**a**) DLIP configuration and (**b**) ITO patterning strategy.

ITO thin films (~ 500 nm thick) deposited on 25 mm × 25 mm × 1.1 mm glass substrates (DELTA Technologies Ltd., USA) were structured by the DLIP technique. Prior to the laser treatment, the surfaces were cleaned using ethanol and dried with compressed air. A DLIP workstation (Fraunhofer IWS, Germany) equipped with a solid-state laser (LXR 100–1030, Luxinar GmbH, Germany) with a maximum output power of 100 W at a pulse frequency of 1 MHz was employed to structure the ITO substrates. The laser operated at a wavelength of ${\lambda}_{\mathrm{L}}=1030 \pm 10\hspace{0.17em}\mathrm{nm}$ with a repetition rate of ${f}_{\mathrm{L}}=50\hspace{0.17em}\mathrm{kHz}$ and a pulse duration of ${\tau }_{\mathrm{L}}=900\hspace{0.17em}\pm 100\hspace{0.17em}\mathrm{ fs}$. The DLIP optical module configuration is shown in Fig. 1a and consists of a telescope system that acts as a beam expander, a diffractive optical element (DOE) that splits the main laser beam into two secondary beams which are then collimated by using a prism and finally focused by a convergent lens onto the surface of the material. The sample was translated using a mechanical stage in two-dimensional directions (x and y) (Aerotech PRO280LM (x-axis) and PRO225LM (y-axis), USA) with a calibrated accuracy of 1 µm, resolution (min. incremental motion) 5 nm and a bidirectional repeatability of ± 0.4 µm. The employed patterning strategy is illustrated in Fig. 1b. Depending on the used pulse-to-pulse distance ${p}_{\mathrm{d}}$ as well as the laser beam diameter 2*ω*_0_, different pulse-to-pulse overlaps $(OV)$ can be achieved as described by the Eq. (1)^41^:

1 $$OV\left[\%\right]=\left(1-\frac{{p}_{\mathrm{d}}}{2{\omega }_{0}}\right)\times 100,$$

By setting the two-beam interference angle to *θ* = 11.82°, the spatial period of the interference pattern was adjusted to $\Lambda =5.0 \hspace{0.17em}\mu \mathrm{m}$, according to the Eq. (2)^42^:

2 $$\Lambda =\frac{{\uplambda }_{L}}{2\mathit{sin}\left(\frac{\theta }{2}\right)},$$

Finally, the hatch distance, which is the lateral distance between successive spots (Fig. 1b), was set to ${h}_{\mathrm{d}}=40\hspace{0.17em}\mathrm{ \mu m}$, while the fluence per pulse was varied from 190 to 340 mJ cm^−2^.

## Characterization methods

To characterize the surface topography of the structured ITO samples, an Atomic Force Microscope was employed (AFM, Nanosurf AG, Switzerland) using the dynamic force mode. The surface profiles and height average values of the topographies were calculated using SensoMAP Advanced Analysis Software (Sensofar, Spain). Besides, Scanning Electron Microscopy (SEM) (Quattro ESEM, Thermo Fischer Scientific, Germany) analysis was performed at an acceleration voltage of 30 kV and the results were analyzed with software Gwyddion^43^.

Total and diffuse transmittances along with total reflectance were obtained using a UV-NIR spectrometer (HR 2000+, Ocean Optics, USA) with an integrating sphere (Thorlabs IS236A-4, Germany).

Finally, electrical characterization of the structured samples was performed with the four-point probe method, employing an electrometer (Sourcemeter 2450, Keithley, USA), and performing two modes of measures, i.e., along (longitudinal) and perpendicular (transversal) to the direction of the pattern. By evaluating the ratio between the measured voltage $V$ and the injected current $I$, a first estimation of the film resistance $R$ was obtained. A correction factor of ~ 0.90 was applied to obtain the appropriate value for the effective sheet resistance $({R}_{\mathrm{S}})$ according to the National Bureau of Standards Technical Note 199 and considering the probe spacing (~ 1.8 mm) along with the size of the sample (~ 5 cm^2^)^44^. This adjustment is needed as the proximity of boundaries might limit the paths of the electrical current in the sample. The values of ${R}_{\mathrm{S}}$ were then approximated using the expression ${R}_{\mathrm{S}}=4.5 \times 0.90 \times R$. Finally, it is worth mentioning that the determined ${R}_{\mathrm{S}}$ corresponds to an effective quantity that ignores the local variations of the film thickness produced after the DLIP process.

## Results and discussion

### Topography evolution upon laser structuring

In order to determine the threshold fluence $({F}_{\mathrm{th}})$ which is necessary to ablate the ITO films, the D^2^ method was employed^45^. This approach allows to calculate this value by measuring the diameter of the modified areas by the laser treatment on the material by firing single laser pulses with different pulse energies. The method yielded values of ${F}_{\mathrm{th}}=180 \pm 10 \hspace{0.17em}\mathrm{mJ }\hspace{0.17em}{\mathrm{cm}}^{-2}$ (with ${\omega }_{0}=115 \pm 1\hspace{0.17em}\mathrm{ \mu m}$, which can be also estimated from this calculation)^45^.

The reported value is comparable with the studies carried out by Kim et al.^46,47^ in which a 150 nm thick ITO film was ablated using pulses with ${\tau }_{\mathrm{L}}=190\hspace{0.17em}\mathrm{ fs}$ at ${\lambda}_{\mathrm{L}}=1030\hspace{0.17em}\mathrm{nm}$, obtaining a threshold fluence value of ${F}_{\mathrm{th}}=140\hspace{0.17em} \mathrm{mJ }\hspace{0.17em}{\mathrm{cm}}^{-2}$. Further threshold fluences obtained by different authors at several process conditions (wavelengths and pulse durations) are described in the supplementary information section (see Table S1)^46–59^.

**Figure 2:**
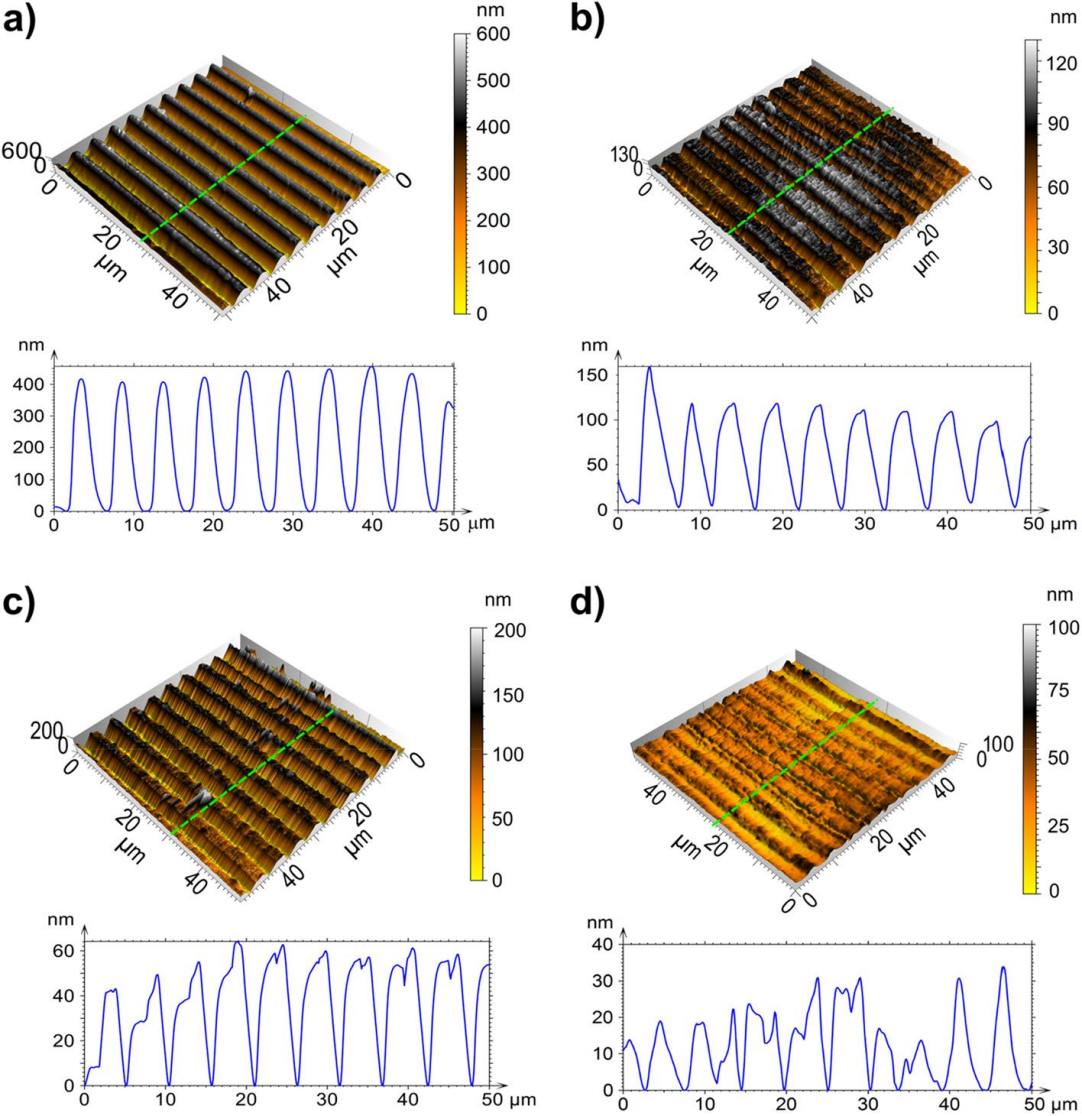
AFM 3D images of laser-structured ITO films along with their height profiles for fluences: (**a**) 340 mJ cm^−2^; (**b**) 250 mJ cm^−2^; (**c**) 220 mJ cm^−2^; (**d**) 190 mJ cm^−2^. The green dashed lines denote where the profiles were taken.

After that, the ITO films were treated using the DLIP method. In Fig. 2(a–d), the topography of DLIP-treated ITO thin films for selected fluence levels (a: 340 mJ cm^−2^; b: 250 mJ cm^−2^; c: 220 mJ cm^−2^; and d: 190 mJ cm^−2^) is displayed. The images were obtained using Atomic Force Microscopy (AFM). In all cases, the pulse-to-pulse overlap (OV) was set to ~ 99% and all samples show a line-like texture with a spatial period of 5.0 µm (corresponding to the spatial period used). For higher values of laser fluence (Fig. 2a,b), well-defined microchannels were obtained with an average height between 100 and 400 nm, whereas for lower values of fluence, less homogeneous microstructures were achieved (Fig. 2c,d). Despite that, the microstructure shown in Fig. 2d was produced at a fluence of 190 mJ cm^−2^, which is slightly above the threshold fluence ${(F}_{\mathrm{th}}=180 \hspace{0.17em}\mathrm{mJ }\hspace{0.17em}{\mathrm{cm}}^{-2}$) the line-like pattern can still be distinguished and an average height of ~ 15 nm can be extracted.

**Figure 3:**
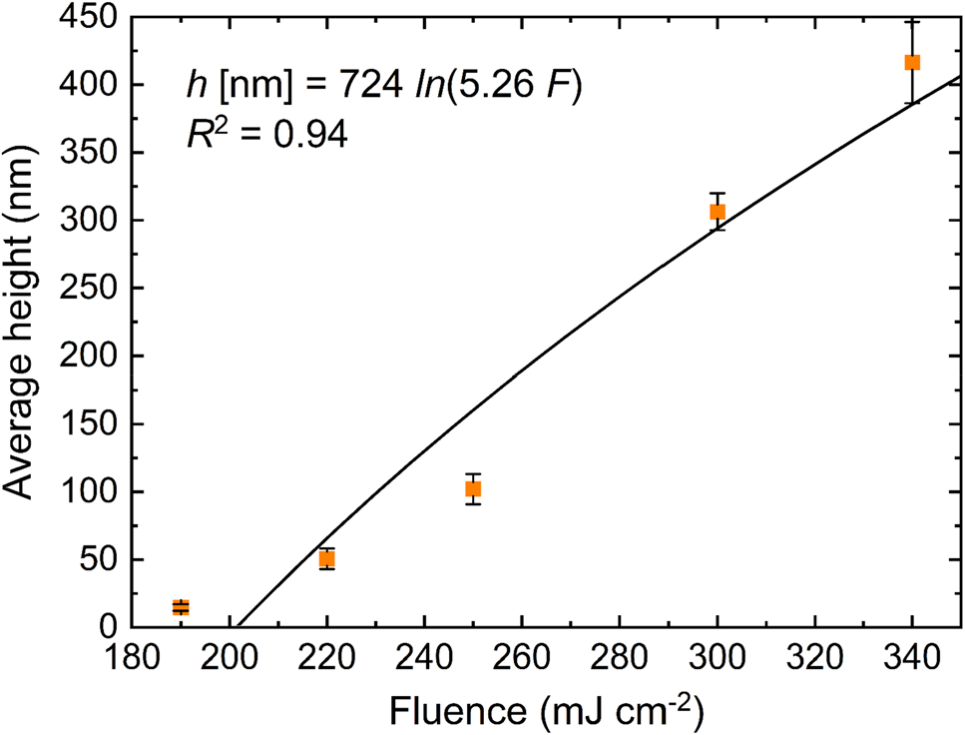
Measured average height of the microstructures as a function of the laser fluence, along with the fit using Eq. (3).

In order to contrast the threshold fluence value calculated previously by the D^2^ method, a logarithmic fit was performed (see Fig. 3) using the measured average height of the microstructures $(h)$ as a function of the applied laser fluence $(F)$, based on the analytical model presented by Byskov-Nielsen et al.^60^ The relationship is described by the Eq. (3):

3 $$h=\frac{1}{\alpha }\mathrm{ln}\left(\frac{F}{{F}_{\mathrm{th}}}\right)$$

This fitting allows determining an approximation of the laser fluence threshold ${F}_{\mathrm{th}}$ of the TCO and the ratio $\frac{1}{\alpha }$ *,* where $\alpha$ is the absorption coefficient. The model is only valid for low fluence levels and considers that the ablation is governed by photochemical ablation processes. Although Eq. (3) was mainly tested on metals interacting with ultra-short laser pulses, in previous studies this approximation was also applied on FTO thin films, obtaining accurate estimations^38,40,61^. Then, the logarithmic fitting curve yielded, ${F}_{\mathrm{th}}=198 \pm 10\hspace{0.17em}\mathrm{ mJ }\hspace{0.17em}{\mathrm{cm}}^{-2}$ and $\frac{1}{\alpha }= 0.72 \pm 0.11\hspace{0.17em}\mathrm{ \mu m}$. On the other hand, the value found for $\frac{1}{\alpha }$ shows a slight discrepancy compared to previous investigations $\left(\frac{1}{\alpha }@1030\hspace{0.17em}\mathrm{ nm }\hspace{0.17em}\sim1 - 2\hspace{0.17em}\mathrm{ \mu m}\right)$^62,63^. A possible explanation for the higher absorption coefficient α found in this study might be related to the non-linear response of ITO towards sub-ps IR laser radiation which might increase the absorptivity. Previous research indicated that the presence of non-linear effects is a phenomenon worth considering on TCO thin films when ultrashort pulsed lasers are used^38,64^. It should be mentioned that the threshold fluence obtained with Eq. (3) is not the single-pulse threshold because the height of the microchannels was obtained using a pulse-to-pulse overlap close to 99%. Nevertheless, the calculated ${F}_{\mathrm{th}}$ with Eq. (3) can still be a reasonable estimation of the single-pulse fluence threshold since the deviation is less than 10% compared to the value determined with the D^2^ method (180 ± 10 mJ cm^−2^) approximation.

**Figure 4:**
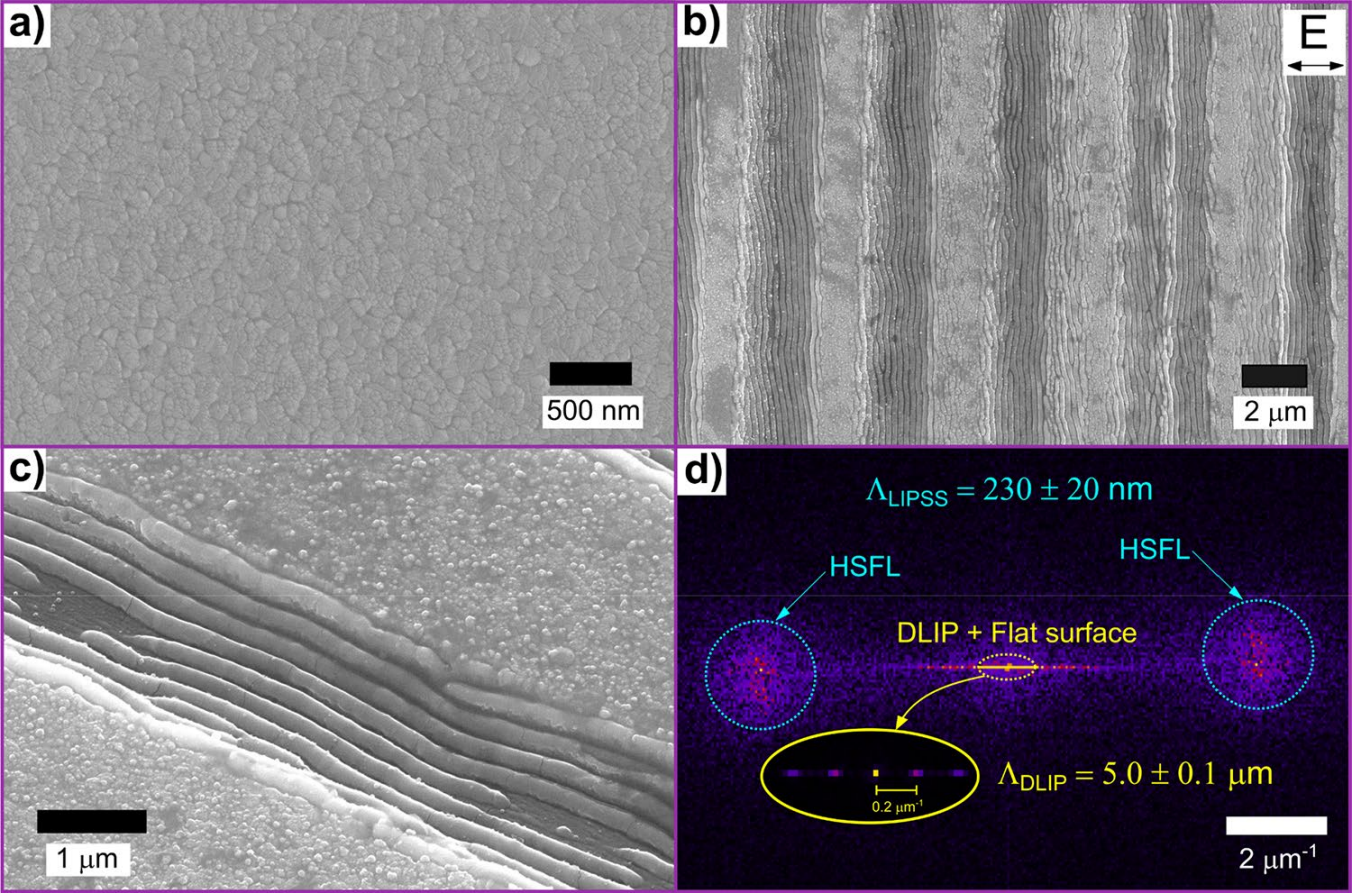
SEM images of (**a**) untreated ITO surface and (**b**) patterned surface with a fluence level of $F=250\hspace{0.17em}\mathrm{ mJ}\hspace{0.17em}{\mathrm{cm}}^{-2}$. The polarization direction of the electrical field is shown with the double arrow. (**c**) Tilted view of the ablated area of the sample shown in (**b**). (**d**) Absolute value of 2D-FFT of Fig. 4b with a zoomed view of the center frequencies. The double-lobes shapes corresponding to High Spatial Frequency LIPSS are denoted by dotted circles.

To study in more detail the morphology of the microgrooves produced by the DLIP treatment, SEM images were taken at different fluence values. Figure 4a shows the SEM image of the untreated ITO surface, whereas Fig. 4b and c are related to the DLIP-treated surfaces with $F=250\hspace{0.17em}\mathrm{mJ}\hspace{0.17em}{\mathrm{cm}}^{-2}$. The untreated sample has a smooth surface characterized by a random distribution of nanocrystallites with a mean size of 42 ± 14 nm. The DLIP-treated sample features a well-defined periodic array of grooves produced by the ablation of ITO at the maxima positions of the interference pattern. For the highest fluence level, a SEM/EDX analysis was carried out to detect the presence of ITO material on the interference maxima positions. The results (see Fig. S1 in the Supplementary Information) suggest that only a small amount of In was detected in the interference maxima, confirming a total ablation of ITO at these positions by DLIP treatment. On the tops of the texture, i.e., at the interference minima positions, the ITO surface does not show any topographical change and similarly to the reference surfaces, presents nanocrystallites with a mean size of 40 ± 12 nm. This suggests that at these locations the laser intensity was not high enough to induce any modification of the films, such as melting, re-crystallization and ablation.

On the valleys of the texture (interference maxima positions), additional features were identified, which can be described as Laser-Induced Periodic Surface Structures (LIPSS)^65^. The LIPSS features are aligned perpendicular to the polarization of the laser (indicated with the double arrow in Fig. 4b). Furthermore, the tilt view displayed in Fig. 4c shows that the LIPSS formed not only on the bottom of the texture but also on the sidewalls. The LIPSS surface is relatively smooth and no crystallites can be identified.

Considering that the pulse duration of the applied pulses was 900 fs and assuming a thermal diffusivity of 0.018 cm^2^ s^−1^, the thermal diffusion length in the ITO material can be approximated to ~ 1 nm^66^. This strongly supports the claim that photochemical ablation (also known as cold ablation) was the process that dictated the laser-matter interaction, which is characterized by negligible heat-affected zones and consequently, not affecting the material at the interference minima positions.

To quantitatively determine the spatial period of the LIPSS features, the two-dimensional Fast Fourier Transform (2D-FFT) was applied to Fig. 4b and its result is displayed in Fig. 4d. From the image, different characteristic elements can be identified. On the one hand, at the central region of the FFT image, well-defined peaks are found to be distributed horizontally, corresponding to the DLIP spatial frequencies (see in detail in the zoomed insert), yielding a spatial period of ${\Lambda }_{\mathrm{DLIP}}=5.0 \pm 0.1\hspace{0.17em}\mathrm{ \mu m}$. On the other hand, the outer area of the figure shows the characteristic double-lobes shapes which are associated with the spatial frequencies of LIPSS^67^. From the position of these features in the image, the spatial period of ${\Lambda }_{\mathrm{LIPSS}}=230 \pm 20\hspace{0.17em}\mathrm{ nm}$ can be calculated.

The observed LIPSS can be classified as High Spatial Frequency LIPSS (HSFL) since their spatial period is smaller than half of the wavelength of the laser source, i.e. $\frac{{\Lambda }_{\mathrm{LIPSS}}}{{\lambda }_{\mathrm{L}}}\cong 0.2< 0.5$^65,68^. This is in agreement with a previous study by Charipar et al.^32^ where nanosecond DLIP was applied on ITO thin films and irregular HSFL were produced perpendicular to the polarization of the electrical field. Such HSFL had a spatial period of 75 nm and considering that a 355 nm laser was used, a ratio (period-to-wavelength) of ~ 0.2 was also obtained. In addition, Bánhegyi et al.^69^ also fabricated HSFL perpendicular to the electrical field on ITO thin films with 150 nm of spatial period by applying 70 fs infrared (1,600 nm) radiation. In this case, the period-to-wavelength ratio was also close to 0.1.

The formation mechanism of HSFL is still under discussion, nevertheless, several approaches such as interference between surface plasmons and the incident wave^70^, local variations of the dielectric constant^71^ and the formation of nano-planes as a consequence of local defects^72^ have been proposed in former studies. Particularly, Farid et al.^73^ and Wang et al.^74^ suggested a reason for the formation of such HSFL on ITO films. In their works, they observed that HSFL were initiated after the growth of nanoblisters with a lateral size of 10–20 nm at the ITO surface, containing fewer O_2_ atoms and thus behaving in a metal-like manner due to the In-In bondings.

**Figure 5:**
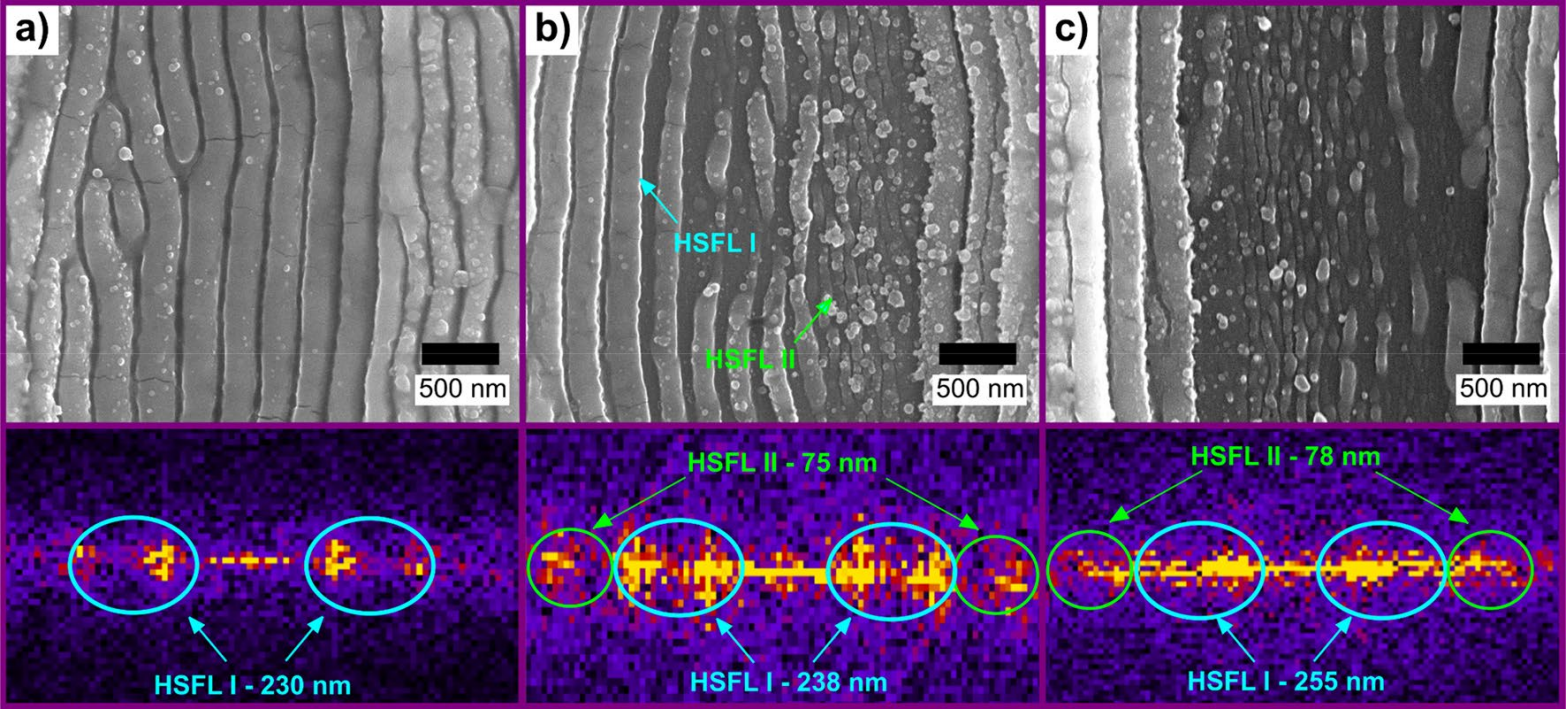
SEM images of DLIP-treated ITO films at an interference maximum, showing different types of LIPSS. The pictures below show the 2D-FFT for fluence levels of (**a**) $F=250\hspace{0.17em}\mathrm{ mJ}\hspace{0.17em}{\mathrm{cm}}^{-2}$; (**b**) $F=300\hspace{0.17em}\mathrm{ mJ}\hspace{0.17em}{\mathrm{cm}}^{-2}$ and (**c**) $F=340\hspace{0.17em}\mathrm{ mJ}\hspace{0.17em}{\mathrm{cm}}^{-2}$. The different types of High Spatial Frequency LIPSS are highlighted with circles (HSFL I and HSFL II).

As the applied laser fluence increased, the formation of a secondary group of LIPSS could be also noticed. This phenomenon is shown in Fig. 5 together with their corresponding 2D-FFT for different fluence levels (a: $250\hspace{0.17em}\mathrm{ mJ}\hspace{0.17em}{\mathrm{cm}}^{-2}$; b: $300\hspace{0.17em}\mathrm{ mJ}\hspace{0.17em}{\mathrm{cm}}^{-2}$ and c: $340\hspace{0.17em}\mathrm{ mJ}\hspace{0.17em}{\mathrm{cm}}^{-2}$). Thus, to differentiate the observed LIPSS according to their feature size, the formed HSFL can be classified as type I and II, as illustrated in the SEM image of Fig. 5b.

In the case of the HSFL type I features, an increase in their average spatial period from 230 to 255 nm was observed as the fluence level underwent from 250 to $340\hspace{0.17em}\mathrm{ mJ}\hspace{0.17em}{\mathrm{cm}}^{-2}$. This dependence between HSFL spatial period and fluence was also reported in former studies in which HSFL were produced with femtosecond pulses in glass, titanium, and carbon fibers^75–77^. On the other hand, the HSFL type II features were mostly generated in the center of the microchannels, i.e., at the interference maxima positions. These features presented a period of approximately 75 nm and since they are three times smaller compared to the HSFL type I features $\left(\frac{{\Lambda }_{{\mathrm{HSFL}}_{\mathrm{I}}}}{{\Lambda }_{{\mathrm{HSFL}}_{\mathrm{II}}}}\sim 3\right),$ it can be argued that they are produced as a consequence of the three-photon absorption (3PA) process, which is a phenomenon commonly observed on TCO materials when treated with ultra-short laser pulses^40,64^.

### Optical characterization

**Figure 6:**
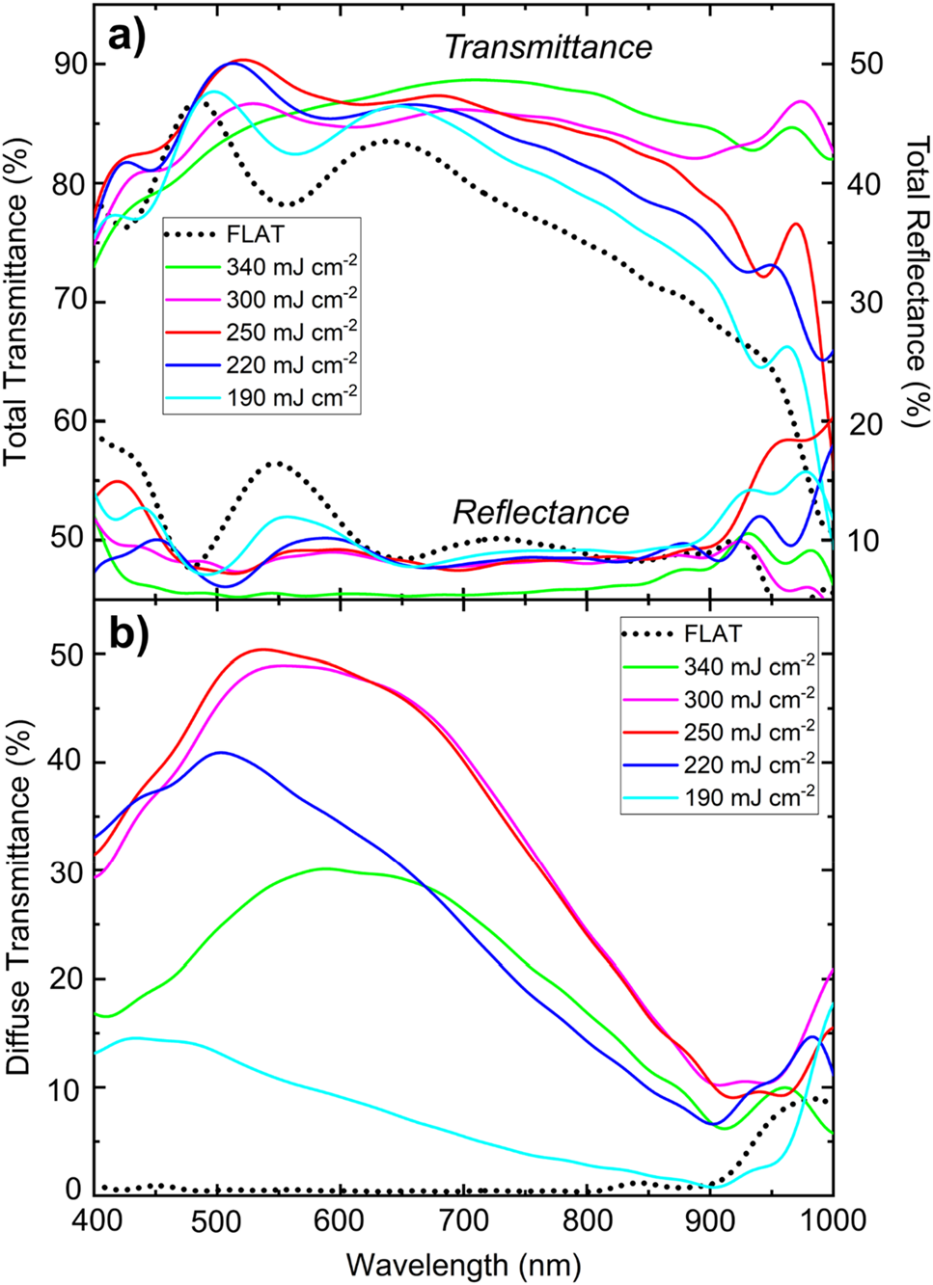
(**a**) Total transmittance, reflectance, and (**b**) diffuse transmittance of structured ITO films for selected fluence values (continuous lines). The dotted lines indicate the values for untreated ITO reference.

To characterize the optical properties of the structured ITO films, the total $({T}_{\mathrm{tot}})$ and diffuse transmittances $({T}_{\mathrm{diff}})$ along with total reflectance $({R}_{\mathrm{tot}})$ in the 400–1000 nm range were measured (Fig. 6). It can be observed that for all samples the total transmittance lies above the reference level for wavelengths longer than 500 nm. In particular, the samples treated with fluence levels of $F=250\hspace{0.17em}\mathrm{ mJ}\hspace{0.17em}{\mathrm{cm}}^{-2}$ and $F=220\hspace{0.17em}\mathrm{ mJ}\hspace{0.17em}{\mathrm{cm}}^{-2}$ have a higher total transmittance compared to the reference sample over the entire spectrum. Furthermore, in the 500–550 nm region, the total transmittance of these samples approached 90%, which is comparable to the total transmittance of high-clarity glass^78^. Such high transmittances can be exploited in photocatalytic devices since the maximum intensity of the solar spectrum is located at a wavelength of approximately 550 nm. In addition, the diffuse transmittance also experienced a remarkable increase reaching absolute values up to 50% for the ITO sample treated at a laser fluence of $F=250\hspace{0.17em}\mathrm{ mJ}\hspace{0.17em}{\mathrm{cm}}^{-2}$ (also at ~ 500–550 nm).

To get an overall estimation of the optical properties of the ITO-treated samples, the average total and diffuse transmittances, total reflectance, and absorbance $(A)$ (defined as *A* = 100% − *T*_tot_ − *R*_tot_) was calculated in the 400–1000 nm spectral range (Table 1). The values indicated in brackets denote their relative variation. It is evident that the enhancement in the total transmittance for all applied fluence levels was a result of a reduction of both, the average reflectance and the total absorbance. The increases in the average ${T}_{\mathrm{tot}}$ and ${T}_{\mathrm{diff}}$ could enhance photon harvesting in photovoltaic devices in the VIS–NIR spectrum or improve light out-coupling in light-emitting diodes^79^.

**Table 1:** Calculated average optical total transmittance ${(T}_{\mathrm{tot}})$, reflectance ${(R}_{\mathrm{tot}})$,, diffuse transmittance ${(T}_{\mathrm{diff}})$, and absorbance $(A)$ in the 400–1000 nm spectral range for each applied fluence.

$F (\mathrm{mJ}\hspace{0.17em}{\mathrm{cm}}^{-2})$	${\overline{T} }_{\mathrm{tot}} (\mathrm{\%})$	${\overline{R} }_{\mathrm{tot}} (\mathrm{\%})$	${\overline{T} }_{\mathrm{diff}} (\mathrm{\%})$	$\overline{A } (\mathrm{\%})$
0 (Ref.)	75.5	10.2	1.6	14.0
190	77.7 (+ 2.6)	10.1 (− 1)	7.7 (+ 380)	12.2 (− 13)
220	80.5 (+ 6.6)	8.9 (− 13)	24.8 (+ 1,450)	10.6 (− 24)
250	82.2 (+ 8.9)	10.0 (− 2)	32.0 (+ 1,900)	7.8 (− 44)
300	83.4 (+ 10.4)	8.2 (− 20)	31.8 (+ 1,880)	8.4 (− 40)
340	83.6 (+ 10.7)	6.4 (− 37)	19.4 (+ 1,100)	10.0 (− 28)

Values in brackets indicate the relative variation with respect to the untreated ITO film.

This change in the optical behavior of the patterned substrates can be attributed to different light-matter interaction mechanisms. Firstly, as a portion of the ITO material was removed through laser ablation, a decrease in the film absorbance is expected. In addition, the formation of the HSFL structures with spatial periods shorter than the wavelengths of visible light act as a material with a gradual effective refractive index, inducing a decrease in the total reflectance. This is in agreement with both experimental and theoretical studies in which a considerable reduction in the total optical reflection was obtained with subwavelength structures on glass^80,81^. Moreover, Solodar et al.^82^ fabricated 145 nm periodic nanostructures on ITO coated glass by fs-laser irradiation and demonstrated an enhancement in transmittance in the visible spectrum attributable to a lower effective refractive index that ultimately reduces reflections at the air/ITO surface.

In addition to the enhanced total transmittance, also a relative increase in the average diffuse transmittance up to 1900% was observed for the ITO films structured at a fluence of $F=250\hspace{0.17em}\mathrm{ mJ}\hspace{0.17em}{\mathrm{cm}}^{-2}$ and $F=300\hspace{0.17em}\mathrm{ mJ}\hspace{0.17em}{\mathrm{cm}}^{-2}$ in comparison to the flat reference. This can be ascribed to the diffraction of incoming light into several diffraction orders induced by the generated periodic microstructures as in a relief diffraction grating^83^. It is expected that the increase in ${T}_{\mathrm{diff}}$ enhances the efficiency of solar cells by improving the light-trapping capability^37^. Namely, as the incident light reaching the front electrode deviates from its original trajectory, the optical path inside the active material becomes larger. Regarding this, Huang et al.^19^ increased the diffuse transmittance in the 300–850 nm spectral region of NiO_x_-coated-hexagonal-tiled textured ITO thin films by ~ 150% (relative) compared to the unstructured reference. This led to an absolute improvement of ~ 1.3% in the efficiency of perovskite-based solar cells, an effect that was not only related to the enhancement of the light absorption but also due to a better hole extraction induced by the increase in the contact area between the electrode and the active layer.

### Electrical characterization

**Figure 7:**
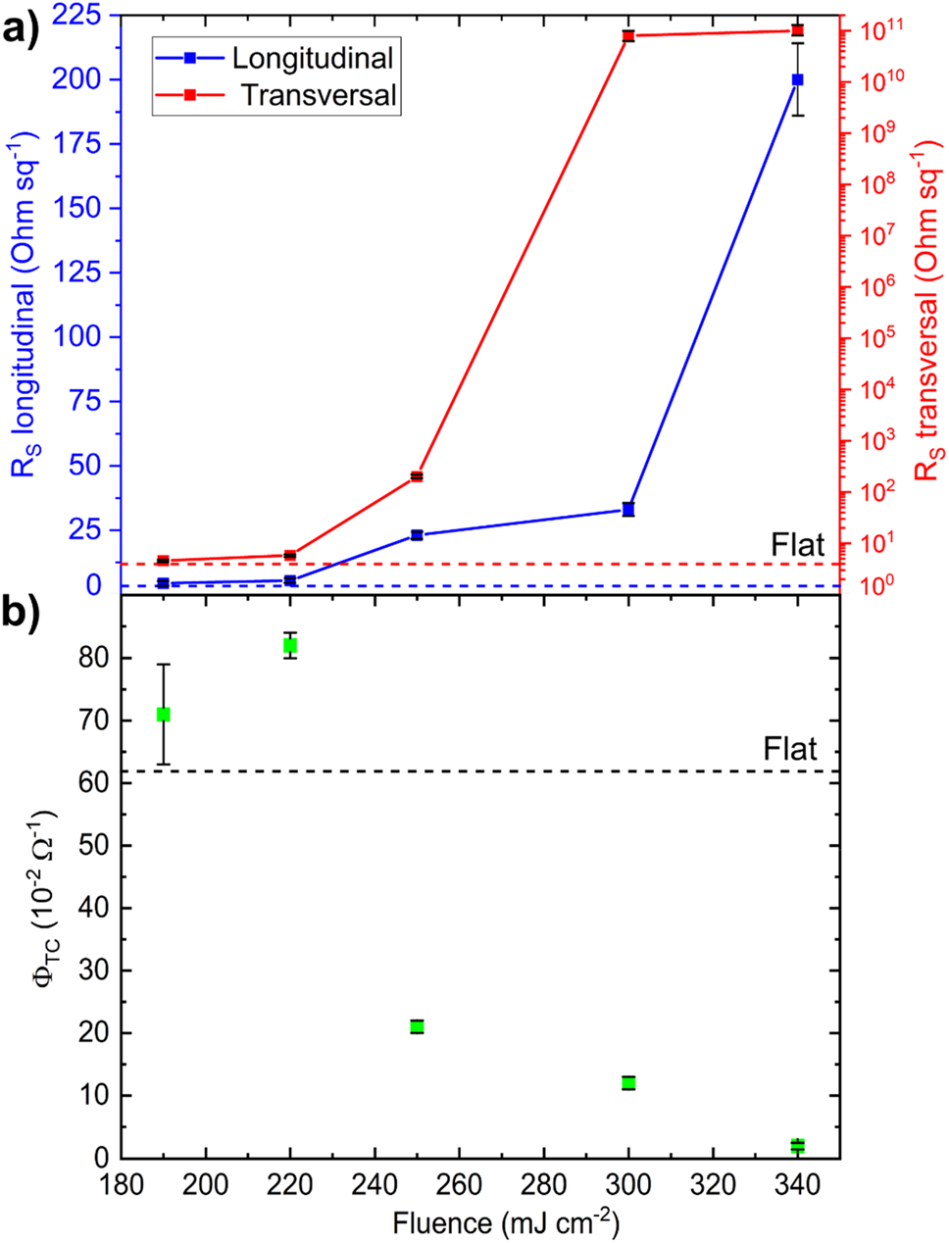
(**a**) Longitudinal effective sheet resistance (blue continuous line), transversal effective sheet resistance (red continuous line) and (**b**) figure of merit $\it {\Phi }_{\mathrm{TC}}$ as a function of the applied laser fluence. The dashed lines indicate the values for untreated ITO.

With the aim of characterizing the change in the electrical behavior of laser-processed ITO, the effective sheet resistance $({R}_{\mathrm{S}})$ as a function of the applied laser fluence was estimated. The results are shown in Fig. 7a for transversal and longitudinal measurement modes (see the section Characterization Methods for details). For a laser fluence of $F=190\hspace{0.17em}\mathrm{ mJ}\hspace{0.17em}{\mathrm{cm}}^{-2}$ an average of the transversal and longitudinal values yields ${R}_{\mathrm{S}}=4.5 \pm 0.2\hspace{0.17em}\mathrm{ \Omega}\hspace{0.17em}{\mathrm{sq}}^{-1}$ which implies a relative increase of 16% compared to unstructured ITO $\left({R}_{\mathrm{S}}=3.8 \pm 0.1\hspace{0.17em}\mathrm{ \Omega}\hspace{0.17em}{\mathrm{sq}}^{-1}\right)$. For fluence levels between 220 and 250 mJ cm^−2^, a rising trend of ${R}_{\mathrm{S}}$ with the laser fluence was observed in both measuring directions as a result of the partial removal of conductive material. Differently, at higher fluences (300 and 340 mJ cm^−2^), a stronger increase of the electrical resistance measured in the longitudinal direction was observed, due to the removal of most of the conductive material at the maxima positions which leads to complete electrical insulation between the ITO ridges. As a result, the ratio between transversal and longitudinal sheet resistances was $\frac{{R}_{\mathrm{STrans}}}{{R}_{\mathrm{SLong}}} \sim 2\times {10}^{9}$ for $F=300\hspace{0.17em} \mathrm{mJ}\hspace{0.17em}{\mathrm{cm}}^{-2}$ and $\sim 5\times {10}^{8}$ for $F=340 \hspace{0.17em}\mathrm{mJ }\hspace{0.17em}{\mathrm{cm}}^{-2}$. This anisotropic behavior was also observed for TCOs patterned with line-like textures using UV DLIP^32^ and IR direct laser writing^84^. Yoshio et al. suggest that this anisotropic characteristic might be suitable for the effective ion transport in electrolytes for catalytic devices such as Li-ion batteries^85^. On the other hand, a former study conducted by Pandey et al.^16^ obtained 10% efficiency on flexible perovskite solar cells by employing a ${R}_{\mathrm{S}}=12\hspace{0.17em}\mathrm{ \Omega}\hspace{0.17em}{\mathrm{sq}}^{-1}$ coated ITO. Moreover, Balestrieri et al.^86^ simulated a heterojunction solar cell with textured ITO as an electrode and their results suggested that improvements can be achieved if the sheet resistance is below 120 Ω sq^−1^ meaning that textured ITO with a laser fluence level below $F=250 \hspace{0.17em}\mathrm{mJ }\hspace{0.17em}{\mathrm{cm}}^{-2}$ might be suitable for solar cell applications.

To analyze the overall performance of transparent conductors (TC) in solar cells, Haacke et al.^87^ introduced a figure of merit ${(\it \Phi }_{\mathrm{TC}})$ as defined by Eq. (4):

4 $$\it {\Phi }_{\mathrm{TC}}=\frac{{\widehat{T}}_{\mathrm{tot}}^{10}}{{R}_{\mathrm{S}}}$$

where ${R}_{\mathrm{S}}$ is the average sheet resistance, and ${\widehat{T}}_{\mathrm{tot}}$ is the average total transmittance in the 400–800 nm range weighted by the solar standard spectral irradiance AM1.5G^88^ using Eq. (5):

5 $${\widehat{T}}_{\mathrm{tot}}=\frac{{\int }_{400\mathrm{nm}}^{800\mathrm{nm}}I{\left(\lambda \right)}_{\mathrm{AM}1.5\mathrm{G}}\times {T}_{\mathrm{tot}}(\lambda )\mathrm{d}\lambda }{{\int }_{400\mathrm{nm}}^{800\mathrm{nm}}I{\left(\lambda \right)}_{\mathrm{AM}1.5\mathrm{G}}\mathrm{d}\lambda }$$

Here, the lower limit of 400 nm was selected due to the limitation imposed by our spectrometer setup, whereas the highest limit of 800 nm coincides with the absorption onset of many thin film absorbers such as polymeric small molecular weight and perovskite semiconductors^89,90^. The results are shown in Fig. 7b, in which the figure of merit ${\it \Phi }_{\mathrm{TC}}$ is illustrated as a function of the applied laser fluence.

The figure of merit for the flat surface is ${\it \Phi }_{\mathrm{TC}}=62\times {10}^{-2} \hspace{0.17em}{\Omega }^{-1}$ which is comparable to the value of ${2\times {10}^{-2}\hspace{0.17em} {\Omega }^{-1} <\Phi }_{\mathrm{TC}}< 50\times {10}^{-2}\hspace{0.17em} {\Omega }^{-1}$ obtained by Beaudry et al.^91^ on ITO with a film thickness of ~ 1 µm. For fluence levels between 190 and 220 mJ cm^−2^, the figure of merit is above the level of unstructured ITO, suggesting a possible improvement in the overall performance of the solar cell when it is treated with the DLIP method for fluence levels near the ablation threshold. For higher fluence levels, the performance is reduced abruptly, since the higher sheet resistance values are dominant compared to the improvements in the optical properties.

## Conclusions

In this contribution, the surface texturing and characterization of ITO thin films using sub-picosecond infrared direct laser interference patterning was presented along with their potential application as a front electrode in solar cells. For lower levels of laser fluence, a slight modification of the surface was obtained characterized by line-like structures with heights between 15 and 70 nm. For higher levels of fluence, well-defined microchannels with a height of up to 450 nm were produced. An exceptional increase (relative) in diffuse and total transmittance up to 1900% and 10.7%, respectively, was achieved as a consequence of the diffraction of incoming light into several diffraction orders induced by the periodic structures (DLIP) combined with a higher light-trapping by sub-wavelength structures (HSFL). The electrical characterization revealed an increase in the effective sheet resistance due to the removal of conductive material and the presence of an anisotropic behavior as a consequence of the formation of well-defined microchannels. Moreover, the empirical figure of merit ${\it \Phi }_{\mathrm{TC}}$ suggests that the optoelectronic performance of solar cells might be enhanced if ITO is structured with an applied laser fluence close to the threshold level. Additionally, it is expected a further efficiency improvement of such devices due to the increase of the effective surface area of the electrode, since this could be suitable for a more effective charge transfer between the electrode and active layer.

Moreover, the surface modifications on ITO shown here could provide additional benefits such as improved light extraction efficiency, customizable work function, increased adhesion properties, and controlled wettability. These improvements can be exploited not only for solar cells but also for next-generation devices, including touchscreens, sensors, and organic light-emitting diodes (OLEDs), where optimized surface properties are essential for achieving superior performance.

## Supplementary Information

Supplementary Information.

## Data Availability

The datasets used and/or analyzed during the current study are available from the corresponding author upon reasonable request.
