# Supplementary material for: Optoelectronic performance of indium tin oxide thin films structured by sub-picosecond direct laser interference patterning
Source: Sci Rep. 2023 Jun 16;13:9798. doi: 10.1038/s41598-023-37042-y (PMC10275878; doi:10.1038/s41598-023-37042-y)
Supplement: Supplementary file 1 — Supplementary Information. [file 41598_2023_37042_MOESM1_ESM.docx]

| **Reference** | **Film thickness [nm]** | **Laser wavelength** $\boldsymbol{(}_{\mathbf{L}}\boldsymbol{)}$ **[nm]** | **Pulse duration** $\boldsymbol{(\tau}_{\mathbf{L}}\boldsymbol{)}$ | **Threshold fluence** $\left( \boldsymbol{F}_{\mathbf{th}} \right)$ **[mJ cm^–2^]** |
| --- | --- | --- | --- | --- |
| This work | 500 | 1030 | 900 fs | 180 ± 10 |
| Park *et al*.^46^ | 200 | 810 | 150 fs | 70 |
| Račiukaitis *et al*.^47^ | 120 | 266 | 10 ps | 210 |
|  |  | 355 |  | 560 |
|  |  | 532 |  | 3040 |
| Choi *et al*.^48^ | 150 | 775 | 150 fs | 700 – 950 |
| Bian *et al*.^49^ | 140 | 800 | 100 fs | 200 |
| Risch *et al*.^50^ | 200 | 355 | <15 ps | 380 |
|  |  | 532 |  | 770 |
|  |  | 1064 |  | 400 |
| Bian *et al*.^51^ | 140 | 800 | 60 fs | 210 |
| Krause *et al*.^52^ | 600 | 1030 | 300 fs | 450 |
| Rung *et al*.^53^ | 90 | 1064 | 10 ns | 1130 |
|  | 110 |  |  | 1050 |
|  | 130 |  |  | 980 |
|  | 150 |  |  | 910 |
| Cheng *et al*.^54^ | 100 | 1064 | < 500 fs | 300 |
| McDonell *et al*.^55^ | 30 | 266 | 8.6 ns | 180 ± 30 |
|  |  | 355 | 20 ns | 320 ± 20 |
|  |  | 532 | 9.0 ns | 3500 ± 490 |
|  |  | 1064 | 9.2 ns | 620 ± 80 |
|  |  | 343 | 500 fs | 250 ± 20 |
|  |  | 1030 |  | 260 ± 30 |
| Kim *et al*.^56,58^ | 150 | 1030 | 190 fs | 140 |
| Yoo *et al*.^57^ | 200 | 1064 | 4.7 ns | 7700 |
| Liao *et al*.^59^ | 300 | 1064 | 13 ns | 1500 |

**Table S1.** Calculated average threshold fluence using the D^2^ method as a function of the film thickness, laser wavelength and pulse duration*.*


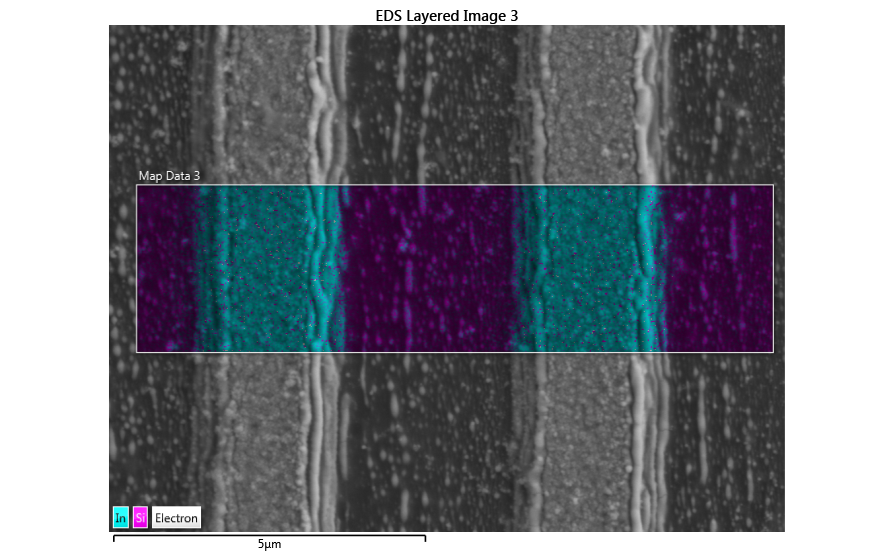


**Figure S1.** SEM/EDX of ITO treated with a fluence level of 340 mJ cm^–2^.
